# Secular Trends in the Prevalence of Overweight and Obesity in Sicilian Schoolchildren Aged 11–13 Years During the Last Decade

**DOI:** 10.1371/journal.pone.0034551

**Published:** 2012-04-10

**Authors:** Cristina Parrino, Paola Rossetti, Roberto Baratta, Nadia La Spina, Lavinia La Delfa, Sebastiano Squatrito, Riccardo Vigneri, Lucia Frittitta

**Affiliations:** Endocrinology Unit, Department of Clinical and Molecular Biomedicine, University of Catania Medical School, Garibaldi Hospital, Catania, Italy; Universita Magna-Graecia di Catanzaro, Italy

## Abstract

The present study evaluates trends in the prevalence of overweight and obesity in relation to gender and area of residence between two cohorts of students aged 11–13 years in Sicily. The analysis was performed on 1,839 schoolchildren, with 924 and 915 children being studied in 1999–2001 and 2009–2010, respectively. The children who were enrolled during 2009–2010 had significantly higher body mass indexes (BMI), BMI z-scores, and waist circumferences than the children who were studied during 1999–2001 (p<0.0001 for all); these differences was also observed when the cohort was subdivided according to gender or residence area The prevalence of obesity increased significantly from 7.9% in 1999–2001 to 13.7% in 2009–2010 (p<0.0001), whereas thinness decreased significantly from 10.1% to 2.3% (p<0.0001) in the same periods. The increase of trends in the prevalence of obesity was significantly higher in males (9.7% vs. 17.6%, p = 0.0006) than in females (6.3% vs. 9.8%, p = 0.04) and was slightly higher in urban areas (8.8% vs. 14.3%, p = 0.002) than in rural areas (7.8% vs. 13.0%, p = 0.012). The male gender was associated with a higher risk of being overweight or obese (odds ratio: 1.63; 95% confidence intervals: 1.24–2.15; p = 0.0005) in 2009–2010 than in 1999–2001, after adjusting for age and the residence area. In conclusion, this study showed an increasing trend in the prevalence of overweight and obesity in Sicilian schoolchildren during the last decade and that this trend was related to gender, age and the area of residence. More specifically, our data indicated that the prevalence of obesity increased by 5.8%, the prevalence of thinness decreased by 7.8% and the prevalence of normal-weight children did not change over the course of a decade. These results suggest a shift in the body weights of Sicilian children toward the upper percentiles.

## Introduction

An increasing prevalence of childhood overweight and obesity [defined as a body mass index (BMI) above the 85^th^ and the 95^th^ percentile for age and gender, respectively] is a serious social and public health problem [1]. Childhood overweight and obesity are especially prevalent in North America and Europe, but they are increasing also in developing countries in Asia, Africa and South America, which are undergoing a rapid nutritional transition [2], [3]. In the last few years, childhood obesity increased in both genders and among all racial, ethnic, and socioeconomic groups, which is a likely consequence of the globalization process that also drove important changes in lifestyle and eating habits [2], [3].

In 2010, 43 million children were estimated to be overweight or obese, and 92 million were at risk of being overweight. The worldwide prevalence of childhood overweight and obesity increased from 4.2% in 1990 to 6.7% in 2010. This trend of obesity prevalence is expected to reach 9.1% of children, or more than 60 million children, in 2020 [4]. As the risk of childhood obesity increases, its health implications (including insulin resistance, hypertension, hyperlipemia, and hepatic steatosis), which are important risk factors for adult morbidity and mortality [3], [5], are also becoming more prevalent in children. Moreover, childhood overweight and obesity are predictive of adult overweight and obesity, with up to 80% of obese children reportedly becoming obese adult individuals [3], [6].

Studies suggest that in the United States, the number of obese children has tripled in the past 20 years [7], but recent data indicate that the prevalence rate of childhood obesity may have reached a plateau [3]. Italy is a European country with a high prevalence of obesity and overweight in children and teenagers, and a geographical trend has been described where childhood overweight and obesity are more prevalent in southern Italy rather than central or northern Italy [2]. In a recent survey of 42,155 eight- to nine-year-old Italian children attending primary school, the overweight prevalence was 22.9% and the obesity prevalence was 11.1% [8]. The highest values were observed in Campania, a southern region of Italy, where 28% of children were overweight and 21% were obese [8]. In Sicily, a southern Italian island, the overweight prevalence was 24% and the obesity prevalence was 13% [8].

In 1999–2001, we observed a high prevalence of overweight and obesity in Sicilian schoolchildren (11–15 year olds) with a cumulative value of nearly 40% at age 11, progressively decreasing with increased age (25% at age 15) [5].

The aim of the present study was to assess the time trends in the prevalence of overweight and obesity, with respect to gender and the area of residence, in a cohort of 11- to 13-year-old Sicilian schoolchildren living in the same areas of the cohort that was studied in 1999–2001.

## Methods

This cross-sectional study is conducted on data recorded from a school-based program during two different periods. The first survey (Phase 1) was conducted from October 1999 to May 2001 by recruiting 48,897 Sicilian schoolchildren [5]. From that cohort, 924 schoolchildren (434 males and 490 females) aged 11–13 years (Table 1) were randomly selected from a population of children that were attending five secondary public schools located in the city of Catania (approximately 350,000 inhabitants on the east coast of Sicily) and in three small country towns (Assoro, Leonforte and Agira, which all had less than 20,000 inhabitants) located in the interior of Sicily that were all classified as rural areas according to ISTAT (Italian Statistical Institute). Phase 2 was conducted from October 2009 through May 2010 and 915 children aged 11–13 years (448 males and 467 females) were recruited from the same public schools that were selected during the first survey (Table 1). In both surveys (1999–2001 and 2009–2010) over 90% of schoolchildren attending the five schools provided parents' consent and entered the study. No exclusion criteria were used. In total, 915 children aged 11–13 years (448 males and 467 females) were recruited from the same schools that were studied during the first survey (Table 1). The study was authorized by the Institutional Board of the Schools, and informed written consent was obtained from the parents for an examination of the children and the anonymous recording of their data.

**Table 1:** The clinical characteristics of the schoolchildren.

	1999–2001	2009–2010	p value
Total number	924	915	
F/M (n)	490/434	467/448	0.392
Urban/rural (n)	486/438	491/424	0.648
Age (years)	12.2±0.8	11.9±0.8	<0.0001
Height (cm)	150.0±0.8	154.0±0.9	<0.0001
Weight (kg)	45.5±11.9	51.6±12.7	<0.0001
BMI (kg/m^2^)	20.0±3.9	21.4±4.1	<0.0001
BMI z-score	−0.1±1.2	0.4±1.1	<0.0001
Waist (cm)	73.7±10.1	77.2±11.3	<0.0001

The data are expressed as the means ± standard deviations.

F = females, M = males, BMI = Body Mass Index.

Children were subdivided by age, according to 1-year intervals, and by gender. A team of 3 trained investigators collected anthropometric (weight and height) measurements. Briefly, the weights (kg) were measured during the morning hours after breakfast with a portable scale with the child in light clothing and without shoes, and the heights (0.1 cm) were measured with a portable stadiometer. The intra- and inter-operator coefficients of variation were calculated with 20 repeats for each operator and were less than 0.1% for both. The body mass index (BMI) was calculated according to the formula weight/height^2^ (kg/m^2^). The BMI z-score was also calculated according to the formula:
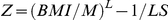
The M and S values correspond to the median and coefficient of variation of body mass index at each age whereas the L value allows for the substantial age dependent skewness in the distribution of body mass index [9].

Waist circumference was measured, after gently exhaling, at the narrowest part between the lower rib and the iliac crest (the natural waist) using a non-elastic flexible tape and recorded to the nearest 0.1 cm.

The ratio between waist and height (W/Hr), both measured in centimeters, was calculated and a cut off of 0.500 used to differentiate low W/Hr from high W/Hr [10], [11].

*Statistical analysis*. All data were cross-sectional for both the study periods (1999–2001 and 2009–2010) and were represented as the means ± standard deviations (SD), unless otherwise stated. Smoothed percentiles for the BMIs were derived using the least mean squares (LMS) method for constructing normalized standard curves, according to the LMS method of Cole et al. [12]. Because the BMI distributions in each age and gender group were not normal and were clearly skewed towards the higher values, the LMS method was used to summarize the BMI distribution at each age, which transforms the positively skewed data into a normal distribution [9]. Children were classified as thin, normal-weight, overweight or obese based on the age- and gender-specific BMI cut-off points for children, which were developed by the IOTF [13], [14]. Thinness was defined as a BMI equal or below the 5^th^ percentile, overweight was defined as a BMI from the 85^th^ to the 94^th^ percentile and obesity was defined as a BMI in the 95^th^ percentile or higher. As the prevalence of obesity was fairly low, the data from children classified as overweight or obese were merged for further analyses. The data were managed with Excel spreadsheets that were double-checked for errors.

The differences between the two period groups (the prevalence at 2009–2010 compared to the prevalence at 1999–2001) were calculated using a chi-squared test for categorical variables and Student's t-test for continuous variables. The annualized change in the prevalence of overweight and obesity was calculated as ([prevalence at 2009–2010 – prevalence at 1999–2001]/10) [15].

Multivariate logistic regression models were applied to assess the risk of being overweight or obese in 1999–2001 compared to 2009–2010. The dependent variable in this model was the binary status of overweight (including obesity), and the study period was the main predictor, with the 2009–2010 study period as the reference category. The analyses were adjusted for age, gender and residence area (urban vs. rural), and the results are presented as adjusted odds ratios (ORs) with 95% confidence intervals (CI). A p value less than 0.05 was considered to be significant.

Data analyses were performed using the SPSS statistical package software (version 15; SPSS Inc., Chicago, IL, USA).

## Results

The analysis was performed in 1,839 schoolchildren that were recruited from the same schools, with 924 children enrolled in 1999–2001 and 915 children in 2009–2010. The examined cohorts (1^st^ and 2^nd^ phase) were not different based on gender (p = 0.392) or their area of residence (urban vs. rural, p = 0.648) (Table 1). The children recruited in 2009–2010 were significantly younger (11.94±0.8 vs. 12.2±0.8 years, p<0.0001), taller (154.0±0.9 vs. 150.0±0.8 cm, p<0.0001), heavier (51.6±12.7 vs. 45.5±11.9 kg, p<0.0001), and had significantly higher BMIs (21.4±4.1 vs. 20.0±3.9 kg/m^2^, p<0.0001), BMI z-scores (0.4±1.1 vs. −0.1±1.2 p<0.0001), and waist circumferences (77.2±11.3 vs. 73.7±10.1 p<0.0001) than the children studied in 1999–2001 (Table 1).

The significant difference that was observed regarding increased BMIs, BMI z-scores, and waist circumferences in the entire cohort in 2009–2010 with respect to 1999–2001 was maintained even when the cohort was stratified according to gender or area of residence (Table 2).

**Table 2:** The trends in the BMIs, BMI z-score, and waist circumferences from 1999–2001 and 2009–2010.

	1999–2001			2009–2010		
Category	BMI (kg/m^2^)	BMI z-score	Waist (cm)	BMI (kg/m^2^)	BMI z-score	Waist (cm)
**Total**	20.0±3.9	−0.1±1.2	73.7±10.1	21.4±4.1*	0.4±1.1*	77.2±11.3*
Females	19.9±3.8	−0.2±1.2	73.4±9.7	21.2±3.9*	0.2±1.0*	76.5±10.5*
Males	20.1±4.1	−0.1±1.2	74.0±10.4	21.7±4.3*	0.5±1.1*	77.9±12.0*
Urban areas	19.7±3.8	−0.2±1.2	72.6±9.8	21.5±4.1*	0.4±1.0*	77.5±11.1*
Rural areas	20.4±4.0	−0.1±1.1	75.0±10.2	21.4±4.2#	0.3±1.1*	76.9±11.6°

The data are expressed as the means ± standard deviations.

BMI = Body Mass Index.

* p<0.0001,

# p<0.005, and

° p<0.01 vs. 1999–2001.

When the changes in BMIs, BMI z-scores, and waist circumferences were analyzed by gender and age distribution, higher values were observed in 2009–2010 for all age groups of both genders, with the exception of 11-year-old girls (Table 3).

**Table 3:** The changes in the BMIs, BMI z-scores, and waist circumferences by gender, age and year of the survey.

	Females			Males		
	11 yrs	12 yrs	13 yrs	11 yrs	12 yrs	13 yrs
**1999–2001**						
BMI (kg/m^2^)	20.4±3.8	19.4±3.7	20.1±3.9	20.1±4.1	19.7±4.0	20.5±4.1
BMI z-score	0.2±1.1	−0.3±1.1	−0.3±1.2	0.2±1.2	−0.2±1.2	−0.2±1.2
Waist (cm)	73.7±9.6	72.0±9.4	74.6±9.9	72.9±10.3	72.9±10.2	75.6±10.6
**2009–2010**						
BMI (kg/m^2^)	20.3±3.8	21.4±3.8*	21.9±4.0*	21.3±4.4#	21.7±4.2*	22.5±4.2*
BMI z-score	0.2±1.1	0.3±1.0*	0.2±1.0*	0.5±1.1#	0.4±1.1*	0.4±1.1*
Waist (cm)	73.4±10.3	77.2±10.5*	79.2±10.0*	76.1±12.4#	77.4±11.0°	80.8±12.4*

The data are expressed as the means ± standard deviations.

* p<0.0001,

# p<0.05, and

° p<0.0005 vs. 1999–2001.

BMI = Body Mass Index, yrs = years old.

We then calculated the W/Hr, an index of abdominal obesity that is believed to be a more accurate tracking indicator of fat distribution and accumulation by age accounting for the growth in both waist circumference and height over age [10], [11]. In 2009–2010 survey, the 43.2% of children had a W/Hr exceeding the 0.5 value against the 38.8% in the first survey (p = 0.054). The percentage of children with W/Hr higher than 0.5 increased significantly in male from 39.4% in 1999–2001 to 47.3% in 2009–2010 (p<0.05) but not in female (38.2% vs. 39.2%, respectively in 1999–2001 vs. 2009–2010).

**Figure 1:**
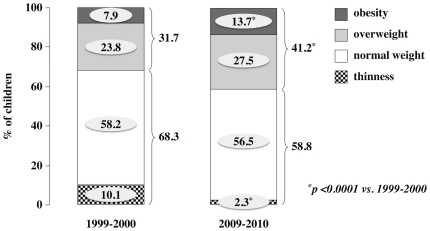
The secular trend in the prevalence of thinness, normal weight, overweight and obesity from 1999–2001 to 2009–2010. During 1999–2001 (left column) and 2009–2010 (right column), 924 and 915 schoolchildren, respectively, were studied; thinness (dotted bars), normal weight (white bars), overweight (light gray bars) and obesity (dark gray bars) were defined according to age- and gender-specific BMI cut-off points for children, as developed by the International Obesity Task Force (IOTF). ^*^p<0.0001 vs. 1999–2000.

In addition, with the IOTF cut-off values, we classified the children as thin (below the 5^th^), normal-weight (between the 5^th^ and the 85^th^), overweight (above the 85^th^ and less than the 95^th^) or obese (equal to or above the 95^th^). The prevalence of thinness significantly decreased from 10.1% in 1999–2001 to 2.3% in 2009–2010 (p<0.0001) (Figure 1). This decrease was significant in both males (from 8.3% to 1.6%, p<0.0001) and females (from 11.6% to 3.0% in 2009–2010, p<0.0001) and among children that resided in urban (from 11.7% to 2.2%, in 2009–2010, p<0.0001) and in rural areas (from 8.2% to 2.4%, p = 0.0001).

During the same decade, the prevalence of overweight increased, although not significantly (p = 0.067), from 23.8% to 27.5% (Figure 1) with no significant difference in either males or females and in children from urban or rural areas. In contrast, the prevalence of obesity significantly increased from 1999–2001 (7.9%) to 2009–2010 (13.7%) (p<0.0001) (Figure 1). The increase in obesity was significantly higher in males (9.7% vs. 17.6%, p = 0.0006) than in females (6.3% vs. 9.8%, p = 0.04) and slightly higher in urban areas (8.8% vs. 14.3%, p = 0.002) than in rural areas (7.8% vs. 13.0%, p = 0.012).

When overweight and obesity were analyzed together, the overall prevalence significantly (p<0.0001) increased during the decade (31.7% vs. 41.2%) (Figure 1) in both males (34.8% to 47.3%, p = 0.0002) and females (29.0% to 35.3%, p = 0.03) and in both urban areas (31.3% vs. 41.5%, p = 0.0009) and rural areas (32.2% vs. 40.8%, p = 0.0086).

In contrast, no significant difference was observed in the prevalence of normal-weight children both in the entire cohort or when divided by gender or area of residence during the decade.

Using a multivariate logistic regression analysis, we observed that the male gender was associated with a higher risk of being overweight or obese (OR: 1.63; 95% CI: 1.24–2.15; p = 0.0005) in 2009–2010 with respect to 1999–2001 after adjusting for age and residence area (urban vs. rural) (Table 4). In contrast, no significant difference in the risk of being overweight or obese was observed in females (OR: 1.24; 95% CI: 0.94–1.63; p = 0.1321) over the decade. Moreover, after adjusting for age and gender, the risk of being overweight or obese was significantly increased in children from both the urban (OR: 1.60; 95% CI: 1.23–2.10; p = 0.0006) and rural areas (OR: 1.41; 95% CI: 1.05–1.90; p = 0.0238) in 2009–2010 with respect to 1999–2001.

**Table 4:** Multivariate logistic regression analysis for the change in the prevalence of overweight and obesity in 2009–2010 compared to that in 1999–2001.

Category	OR (95% CI)	p value
Females	1.24 (0.94–1.63)	0.132
Males	1.63 (1.24–2.15)	≤0.0005
Urban areas	1.60 (1.23–2.10)	<0.001
Rural areas	1.41 (1.05–1.90)	<0.05
**Overall**	1.42 (1.17–1.73)	<0.0005

OR = odds ratio; 95%.

CI = confidence intervals.

All models are adjusted for the age, sex and area of residence.

Overall, after adjusting for age, gender and residence area, the risk of being overweight or obese was significantly increased in 2009–2010 (OR: 1.42; 95% CI: 1.17–1.73; p = 0.0004) than in 1999–2001.

## Discussion

Our study shows a significant rise in the prevalence of BMI, BMI z-score, waist circumferences and of overweight and obesity in male and female Sicilian schoolchildren aged 11–13 years during the last decade. This trend was observed with respect to gender, age and area of residence. During the same decade, a significant decrease in the prevalence of thinness was observed for both genders and the areas of residence. Our results are of particular concern, because in a ten-year period Sicilian schoolchildren, especially males, have become alarmingly heavier and fatter, with an unfavorable fat distribution.

Our data indicate that more than 47% of male and 35% of female Sicilian schoolchildren that were 11–13 years old were at or above the 85^th^ percentile of BMIs for age and gender in 2009–2010. In the last decade, the prevalence of obesity increased more in males (decade increase 7.9% or an annual increase of 0.79 percentage points) than in females (decade increase 3.5% or an annual increase of 0.35 percentage points), reflecting the higher prevalence of overweight and obesity that has been also reported in males from other countries [16]–[20]. The social and/or biological basis of this gender difference is currently unknown.

Childhood obesity is a relevant problem that is emerging worldwide [16]. The upward trend in the prevalence of overweight and obesity during the past two or three decades has been observed in most industrialized countries and in several low-income countries, especially in urban areas [21]. The prevalence doubled or tripled from the early 1970s to the late 1990s in Australia, Brazil, North America (Canada and USA) and Europe (France, Germany, Greece, Finland, and UK) [21]. However, recent data, which is still preliminary, suggest that the increase in childhood obesity has reached a plateau or even might be abating in the USA, the UK and Sweden [22]–[25]. The BMI distribution among adults and children in the USA has become increasingly skewed to the right, indicating an increased prevalence of obesity, and the stabilization of obesity rates among children and adolescents (as revealed in the NHANES data) does not extend to the heaviest boys (those with a BMI at or above the 97^th^ percentile for their age) whose numbers continue to increase [26].

Compared with these data, the overweight and obesity prevalence is one of the highest ever reported for Sicilian schoolchildren both ten years ago [5] and currently [27], [28]. By 2010, obesity was expected to be 10% and 20% among European children and adults, respectively. In the current study, the mean prevalence of obesity in Sicilian children was higher than the expected value, suggesting a greater annual increase in obesity [28]. In fact, our data indicate a decade increase of 5.8% for the prevalence of obesity, a 7.8% decrease in the prevalence of thinness and no change in the prevalence of normal-weight children, which suggests a shift of body weight in the Sicilian children's population toward the upper percentiles.

These observations are similar to, but more dramatic than, the data published in 2006 and reported in the late 1990s concerning an annual increase in the overall prevalence of overweight and obesity of approximately 1.0% and of 0.3%, respectively, for obesity alone [29].

In Italy, a recent study [30] indicates that the prevalence of obesity among children of the same age as those studied in our survey is much lower in Tuscany (a north-central region of Italy) in both males (3.4% at 11 years and 3.3% at 13 years) and females (3.2% at 11 years and 3.2 at 13 years) and that the trend was stable in the short interval between 2004 and 2006.

The cause of this striking difference between two areas of Italy, which have a similar lifestyle, is not clear, and our study does not provide an explanation. Most likely, obesity increases more in Sicilian children because of an interaction between biological (genetic) and social (environmental and lifestyle) factors, which may include a lower average family income, reduced physical activity opportunities, and an overabundance of high-density caloric food. The recent (last 3 decades) change in the eating habits observed in Sicily reveals a strong increase in the availability of industrial and processed foods that are high in calories and an increasing number of meals that are eaten away from home, even in rural areas where the use of autochthon foods was typical in the past. Thus, we can speculate that this change can contribute to the observed increase of obesity prevalence. The additive effects of genetic and environmental variance are most likely associated with the prevalence of overweight and obesity and the mean BMI distribution. An obesity-promoting environment enhances the effects of genes related to adiposity via regulation at a genetic level or because of more distal environmental restrictions on gene expression [31].

Another possible reason for the difference between the two regions of Italy is the collection method. Data on the Tuscany adolescents were self-reported. It is known that people often claim to be taller and thinner than they actually are, which suggests that an obesity prevalence based on self-reported heights and weights can be underestimated [26]. In any case, these observations should be a matter of concern for health authorities for two reasons: first, a growing number of obese children now have diseases that were once considered to be typical of adults (type 2 diabetes, nonalcoholic fatty liver disease, and hypertension), and second, an earlier age of onset of obesity-related diseases may be associated with more severe health consequences in adulthood [26].

Additionally, a novel observation is the decreasing prevalence of thinness in both male and female schoolchildren with no differences between the urban or rural areas. While extensive data are available on the frequency of childhood overweight or obesity, few data are available on the prevalence of thinness in industrialized countries. A cross-sectional study showed a trend for an increasing prevalence of thinness across different ages (7–9 years) in French children [32], while an Italian study indicated a slight reduction during a short period (from 2004 to 2006) in the thinness prevalence particularly in males, which are data that are in agreement with our findings [30].

Body-fat mass and distribution influence the risk of type 2 diabetes and cardiovascular disease among adults and abdominal obesity is associated with metabolic disorders among children and adolescents [33]. Waist circumference and W/Hr are two simple, yet effective surrogate measures of abdominal obesity in adults and children [10], [34].

Our data, indicating a mean waist circumference increase of 3.1 cm among female children and 3.9 cm among male children in the last decade and an increased proportion of male children exceeding the 0.500 W/Hr are in agreement with the secular trend reported in children of other Countries [34].

In conclusion, we report that the prevalence of obesity increased significantly in the last decade among adolescents aged 11–13 years in Sicily. In addition, our results indicate that the prevalence of a high BMI and waist circumference during childhood is increasing strikingly in Sicily with a general shift towards higher BMIs in the population of children and that the male gender is associated with a significant risk of obesity.

More research is urgently needed to identify the behavioral, biological, and environmental factors that cause the prevalence of abnormally high BMIs in the Sicilian children. A high BMI is an abnormality that will cause an epidemic of adult obesity and related complications with a well-known social and economic burden in the future.
